# Interprofessional communication in medical simulation: findings from a scoping review and implications for academic medicine

**DOI:** 10.1186/s12909-022-03226-9

**Published:** 2022-03-26

**Authors:** Sadie Trammell Velásquez, Diane Ferguson, Kelly C. Lemke, Leticia Bland, Rebecca Ajtai, Braulio Amezaga, James Cleveland, Lark A. Ford, Emme Lopez, Wesley Richardson, Daniel Saenz, Joseph A. Zorek

**Affiliations:** 1 Joe R. and Teresa Lozano Long School of Medicine, University of Texas Health Science Center at San Antonio, San Antonio, TX USA; 2 South Texas Veterans Health Care System, Medicine Service, San Antonio, TX USA; 3 Department of Medicine, Division of Hospital Medicine, University of Texas Health Science Center at San Antonio, 7703 Floyd Curl Drive, San Antonio, TX 78229 USA; 4 H-E-B Clinical Skills Center, University of Texas Health Science Center at San Antonio, San Antonio, TX USA; 5 School of Dentistry, University of Texas Health Science Center at San Antonio, San Antonio, TX USA; 6 School of Health Professions, University of Texas Health Science Center at San Antonio, San Antonio, TX USA; 7 Briscoe Library, University of Texas Health Science Center at San Antonio, San Antonio, TX USA; 8 School of Nursing, University of Texas Health Science Center at San Antonio, San Antonio, TX USA; 9 Center for Simulation Innovation, University of Texas Health Science Center at San Antonio, San Antonio, TX USA; 10 Graduate School of Biomedical Sciences, University of Texas Health Science Center at San Antonio, San Antonio, TX USA; 11 Linking Interprofessional Networks for Collaboration (LINC), Office of the Vice President for Academic, Faculty & Student Affairs, University of Texas Health Science Center at San Antonio, San Antonio, TX USA

**Keywords:** Interprofessional education, Interprofessional communication, Medical education, Simulation

## Abstract

**Background:**

Interprofessional communication is fundamental to the delivery of healthcare and can be taught in medical school and other health professional schools through interprofessional education (IPE) activities. Simulation centers have become a predominant location for simulation IPE activities with infrastructure able to support high fidelity activities in a controlled environment. In this secondary analysis of a scoping review conducted on simulation-based IPE, we describe the characteristics of previously reported simulation IPE activities involving undergraduate medical students in a simulation center focused on interprofessional communication.

**Methods:**

Electronic searches of PubMed, CINAHL, and ERIC databases in accordance with PRISMA-ScR guidelines were conducted to isolate relevant articles from 2016–2020. In total, 165 peer-reviewed articles met inclusion criteria and data extraction linked to four research questions was applied by one individual and the accuracy was confirmed by a second individual. A secondary analysis was performed to describe what existing approaches for simulation IPE in simulation center settings have been used to explicitly achieve interprofessional communication competencies in undergraduate medical education. A sub-dataset was developed from the original scoping review and identified 21 studies describing simulation IPE activities that took place in dedicated simulation centers, targeted the IPEC interprofessional communication domain, and involved undergraduate medical students.

**Results:**

Though diverse, the majority of simulation IPE activities described high-fidelity approaches involving standardized patients and utilized assessment tools with established validity evidence in IPE activities to measure learning outcomes. A minority of simulation IPE activities were described as hybrid and utilized more than one resource or equipment for the activity and only two were longitudinal in nature. Learning outcomes were focused predominantly on modification of attitudes/perceptions and few targeted higher levels of assessment.

**Conclusions:**

Educators charged with developing simulation IPE activities for medical students focused on interprofessional communication should incorporate assessment tools that have validity evidence from similar activities, target higher level learning outcomes, and leverage hybrid models to develop longitudinal simulation IPE activities. Though an ideal environment to achieve higher level learning outcomes, simulation centers are not required for meaningful simulation IPE activities.

**Supplementary Information:**

The online version contains supplementary material available at 10.1186/s12909-022-03226-9.

## Introduction

Interprofessional communication is defined by the Interprofessional Education Collaborative (IPEC) as ‘the ability to communicate with patients, families, communities, and professionals in health and other fields in a responsive and responsible manner that supports a team approach to the promotion and maintenance of health and the prevention and treatment of disease’ and is one of the four IPEC competencies [1]. Interprofessional communication aids in preparing health professionals for collaborative practice, allowing them to communicate their readiness to work together [1]. Ineffective interprofessional communication amongst health professionals leads to errors, delayed treatment, misdiagnosis, and adverse events, all of which result in poor patient outcomes [1–5]. Communication failures are reported to be the cause of 56% of intraoperative and postoperative complications [2]. The need for improved communication is a priority in healthcare and the World Health Organization promotes interprofessional healthcare learning as an approach to address communication to improve patient safety and patient outcomes [1, 6, 7]. Improved interprofessional communication and training in clinical settings results in fewer reported adverse events, fewer counting errors in the operating room, improved performance, and probable savings in healthcare costs [3, 8]. In intensive care settings, improved communication during interprofessional rounds has been shown to decrease adverse events by enhanced interprofessional teaching and coordination of patient care [9].

Accrediting bodies throughout the health professions, such as the Liaison Committee on Medical Education (LCME), Commission on Dental Accreditation, Commission on Collegiate Nursing Education, and the Accreditation Council for Pharmacy Education, along with their counterparts from the Health Professions Accreditors Collaborative, recognize that communication failures amongst health professionals is a problem. As a result, a large proportion of accreditors have included requirements for interprofessional communication-focused education in their standards and guidelines [10–15]. The LCME, which maintains accreditation authority for medical colleges and schools in the United States and Canada, emphasizes the importance of interprofessional education (IPE) by including a requirement that core curriculum within medical schools must prepare students to function collaboratively on teams inclusive of other health professionals with specific instruction in interprofessional communication and interprofessional care [10].

Instruction devoted to interprofessional communication is common and is being incorporated into health professionals’ education using a variety of approaches, including online modules, case studies, workshops, and simulations [16, 17]. Interprofessional communication workshops with clinical scenarios using communication tools such as Strategies and Tools to Enhance Performance and Patient Safety curriculum (TeamSTEPPS™) have shown to increase confidence and perceived competence in conflict resolution [17–19]. Training in small group role-play of clinical cases, small group discussions, and presentations positively improved scores on the Global Interpersonal Communication Competence Scale (GICC-15), a tool with established validity evidence for measuring communication among health professionals, showing that students’ overall communication competency can increase with practice [20]. Communication courses are another highly utilized means to positively impact health professional learners’ interpersonal and interprofessional communication self-efficacy beliefs [21, 22]. Hagemeier et al. described an interprofessional communication development skills course for pharmacy, nursing, and medical students that improved students’ interpersonal and interprofessional communication self-efficacy beliefs (i.e., belief in one’s ability to succeed in a situation) after an eight-module course [21].

Simulation has been shown to be one of the most effective forms of health professions education [23]. Opportunities exist to leverage the effectiveness of simulation to advance learning objectives focused on interprofessional communication to meet LCME standards and achieve interprofessional communication-focused IPEC competencies [16, 23]. Liaw et al. combined a standardized patient (SP) and patient simulator for a clinical scenario to provide variation and heightened realism for an interprofessional team in the case of a deteriorating patient, showing increased confidence in communication for health professional learners [24]. A SP is defined as “a person trained to consistently portray a patient or other individual in a scripted scenario for the purposes of instruction, practice, or evaluation” [25]. Standardized patients are commonly used to teach communication skills and their use has been shown to lead to significant increases in self-reported communication skills [19, 26–28]. Fidelity in simulation is defined as “the degree to which the simulation replicates the real event and/or workplace; this includes physical, psychological, and environmental elements” [29]. High-fidelity simulation refers to extremely realistic simulation experiences that provide a high level of interactivity and realism for the health professionals [29, 30]. Simulation centers are widespread and growing in health professions education, providing an opportunity to develop large-scale IPE simulations that involve an optimized number and combination of students at the appropriate level of training for interprofessional practice [24, 31, 32]. These settings, also referred to as clinical skills centers, are commonly used as a location for high-fidelity simulation specifically with SPs and SP training programs, leveraging dedicated staff and examination rooms in a controlled environment in medical education [33, 34]. The Association of American Medical Colleges reports that increased activities for medical students in a simulation center result in increased repetitive opportunities for learning clinical skills and assessment throughout the medical curriculum [35]. Simulation centers have been used to develop early interprofessional teamwork and communication skills in medical and nursing students which may possibly affect professional practice and subsequent patient safety [24].

Despite growing expectations across health professions education for longitudinal IPE, IPE activities occur most frequently as single events, thus limiting the goals of modifying health professionals’ behaviors and potentially improving patient outcomes [14, 15, 36]. The importance of longitudinal studies, commonly defined as taking place over at least one year, has been recognized [37]. Longitudinal IPE studies have shown improvement in perceptions and skills amongst health professional learners but are lacking in number [36, 38–40].

The introduction of interprofessional communication in simulation activities in undergraduate medical education may lead to improved interprofessional communication beyond medical education, though further descriptions of these simulation activities is needed. In this secondary analysis of a scoping review conducted on simulation-based IPE, we aimed to determine the characteristics of previously reported simulation IPE activities involving undergraduate medical students in a simulation center that explicitly identified increasing competence in interprofessional communication as a desired outcome. We sought to identify co-learners, other targeted IPEC competencies, and intended learning outcomes using the Kirkpatrick’s Expanded Outcomes Typology [41]. We also sought to compile practical considerations reported by authors, including barriers to successful execution.

## Methods

A complete description of our scoping review protocol, conducted in accordance with JBI guidelines for scoping reviews (i.e., PRISMA-ScR), has been previously published [42]. In summary, we searched PubMed directly and used the EBSCO platform to search CINAHL Plus and ERIC databases with results limited to the year 2016. Our scoping review was designed to inform the following research questions: In what contexts has simulation IPE been implemented, in regard to learner type, setting, topics, level of fidelity, and resources used? Which IPEC competencies for interprofessional collaborative practice have been included in the design of simulation IPE? What student learning, educator-related, and patient-related outcomes have been measured and reported in the design of simulation IPE? What are the facilitators and barriers to simulation IPE?

These research questions were intentionally broad in order to gather a general and expansive view of the literature. Inclusion criteria included study participants, concept, context and types of data sources. Studies published in peer-reviewed literature that reported on simulation activities involving two or more types of undergraduate or graduate health professional learners in the simulation environment that were delivered in academic settings, clinical settings, community settings, or other settings and included either quantitative, qualitative, or mixed methods were included.

In total, 165 peer-reviewed articles met inclusion criteria, and a 31-item data extraction tool linked to our four research questions was applied. The complete data extraction tool has been previously published with the protocol; examples of data extracted included types of learners (by profession), settings, IPEC competencies targeted, learning outcomes, and reported assessment/evaluation strategies [42]. Data extraction was completed by an individual team member. A second team member independently reviewed and confirmed the accuracy of extracted data. Identified discrepancies were resolved by these individuals with a third team member available to adjudicate unresolved discrepancies.

Kirkpatrick’s Expanded Outcomes Typology was used to categorize learning outcomes, commonly utilized in educational training and simulation as a method of evaluating and categorizing outcome criteria of educational training [41, 43, 44]. Kirkpatrick’s original four-level model classifies learning into reaction, learning, behavior, and results [44]. This was later expanded with an explicit aim of application to IPE, as a mechanism to demonstrate the opportunities for IPE across the learning continuum as learners move from preclinical curricula and clinical learning environments into practice. The Kirkpatrick model includes levels and sublevels [41, 45]:

- Level 1: Learner’s reaction
- Level 2a: Modification of attitudes/perceptions
- Level 2b: Acquisition of knowledge/skills
- Level 3: Behavioral change
- Level 4a: Change in organizational practice
- Level 4b: Benefits to patients, families, and communities

Additional research questions were developed to guide secondary analyses of the complete scoping review dataset. This secondary analysis was conceived to inform strategies to address LCME accreditation requirements and was organized around the following research question: What existing approaches for simulation IPE in simulation center settings have been used to explicitly achieve interprofessional communication competencies in undergraduate medical education? Guided by this question, a sub-dataset was developed from the original by identifying studies describing simulation IPE activities that (1) took place in dedicated simulation centers, (2) targeted the IPEC interprofessional communication domain, and (3) involved undergraduate medical students.

## Results

Of the 165 articles that met inclusion criteria for our scoping review, 144 were excluded from the secondary analysis because they did not meet all three criteria; therefore, 21 articles published between 2016–2020 were included (Table 1). Simulation IPE activities covered a variety of topics, including mental health, cardiovascular health, oral health, medication errors, end-of-life care, discharge planning, inpatient and outpatient care, transitions of care, and emergency care. The majority of simulation IPE activities were categorized as high-fidelity (76%, 16/21). Standardized patients were utilized in 71% (15/21) of activities; 29% (6/21) utilized mannikins only. Thirty-three percent (7/21) were categorized as hybrid activities, utilizing more than one resource or equipment for the activity, to include virtual reality, mannikins, simulated health records, and SPs. Ten percent (2/21) were longitudinal simulation IPE activities. Medical students most commonly worked with nursing students (undergraduate and graduate) in identified simulation IPE activities (90%, 19/21), followed by pharmacy students (43%, 9/21). Less commonly reported were partnerships with occupational therapy and physical therapy (19%, 4/21); dentistry and dietetics/nutrition (each 14%, 3/21); physician assistant, social work, and speech language pathology (each 10%, 2/21); midwifery, operating department practitioner, psychology, public health, and respiratory therapy (each 5%, 1/21).

**Table 1:** Simulation-based Interprofessional Education Activities from 2016–2020 with a Focus on Interprofessional Communication with Undergraduate Medical Learners

Author and Year of publication^a^	Article Title	Description of activity	Participant type, professions represented^b^
Reising et al 2017 [46]	Team Communication Influence on Procedure Performance: Findings from Interprofessional Simulations with Nursing and Medical Students	Teams of students took part in a range of interprofessional activities including team trainings, standardized patient interactions, and direct practice during a longitudinal study	Medicine Nursing (undergraduate)
Oxelmark et al 2017 [47]	Students’ Understanding of Teamwork and Professional Roles after Interprofessional Simulation—A Qualitative Analysis	Students participated in a one-day training to strengthen the competencies needed for collaborative management of emergency situations	Medicine Nursing (undergraduate)
Partecke et al 2016 [48]	Interprofessional Learning – Development and Implementation of Joint Medical Emergency Team Trainings for Medical and Nursing Students at Universitätsmedizin Greifswald	Students participated in a two-day simulation-based course module in clinical emergency medicine	Medicine Nursing (undergraduate)
Horsley et al 2016 [49]	Developing a Foundation for Interprofessional Education Within Nursing and Medical Curricula	Students completed TeamSTEPPS education prior to participating in two simulation interprofessional education activities. After the first activity, students were debriefed and TeamSTEPPS principles were reinforced prior to the second simulation activity. Pre and posttest evaluations were performed	Medicine Nursing (undergraduate)
Jakobsen et al 2018 [50]	Examining Participant Perceptions of an Interprofessional Simulation-based Trauma Team Training for Medical and Nursing Students	Students participated in four simulation activities alternating with interactive lecture sessions. Debrief sessions followed the activities and a questionnaire was developed based on student learning outcomes in two phases. Data was also collected from faculty facilitators in the third phase	Medicine Nursing (graduate—unspecified)
Sincak et al 2017 [51]	Transformation of an Online Multidisciplinary Course into a Live Interprofessional Experience	Students were placed in interprofessional groups, meeting weekly for ten weeks in a required course. Students participated in didactic lectures, discussion sessions, and a standardized patient encounter. The objectives were to learn about the roles and responsibilities of each profession, teamwork, and how to improve collaboration and team-based communication skills	Dentistry (unspecified) Medicine Occupational Therapy Pharmacy Physical Therapy Physician Assistant Psychology (unspecified) Speech-Language Pathology
Reed et al 2017 [52]	Simulation Using TeamSTEPPS to Promote Interprofessional Education and Collaborative Practice	The TeamSTEPPS curriculum was used as the foundation of an educational intervention for students along with simulated learning activities. The intervention focused on what type of training and how succinct training should be for effective influence on students’ knowledge, self-efficacy, and overall team performance	Medicine Nursing (undergraduate)
Sehgal et al 2019 [53]	First Do No ‘Pharm’: Educating Medical and Pharmacy Students on the Essentials of Medication Management	Students participated in a multi-part interprofessional education session on medication management involving a pillbox exercise and a medication reconciliation exercise	Medicine Pharmacy
Andersen et al 2018 [54]	Interprofessional Simulation: Developing Teamwork Using a Two-Tiered Debriefing Approach	The TeamSTEPPS Team Performance Observation Tool was used to guide this activity. Authors observed, surveyed, and interviewed participants to evaluate the impact of a two-tiered debrief approach on medical, physiotherapy, and nursing students learning interprofessional communication and teamwork	Medicine Nursing (undergraduate) Physical Therapy
Shrader et al 2016 [55]	Using Communication Technology to Enhance Interprofessional Education Simulations	Students participated as part of a required capstone course. Students were randomly assigned to one of three interprofessional education simulations with other health professional students using communication methods such as telephone, e-mail, and video conferencing	Dietetics and Nutrition Medicine Nursing (undergraduate) Nursing (DNP) Occupational Therapy Pharmacy
Ragucci et al 2016 [56]	Evaluation of Interprofessional Team Disclosure of a Medical Error to a Simulated Patient	Students participated in simulated interprofessional rounding experiences and completed a workshop to improve their confidence and proficiency in disclosing medical errors to patients	Medicine Nursing (undergraduate) Pharmacy Physician Assistant
Kusnoor et al 2019 [57]	An Interprofessional Standardized Patient Case for Improving Collaboration, Shared Accountability, and Respect in Team-Based Family Discussions	Students worked in teams to disclose a medical error to a standardized patient	Medicine Nursing (undergraduate) Pharmacy
Carpenter et al 2018 [58]	Interprofessional Collaborative Practice: Use of Simulated Clinical Experiences in Medical Education	Students participated in a clinical scenario with mannikins presenting with common conditions. Student teams took a history, performed an examination, and reported findings to the patient and attending physician	Medicine Nursing (undergraduate)
Wen et al 2019 [59]	An Interprofessional Team Simulation Exercise About a Complex Geriatric Patient	Students collaborated in a simulation to develop a discharge plan for a patient and discuss the plan with the patient's family in a family meeting. A retrospective pre/post survey was performed and qualitative and quantitative analyses were performed	Medicine Nursing (undergraduate) Pharmacy Public Health Social Work
Liaw et al 2020 [60]	“Getting Everyone on the Same Page”: Interprofessional Team Training to Develop Shared Mental Models on Interprofessional Rounds	Students participated in simulations involving a patient showing symptoms of sepsis and/or septic shock. Participants were randomized to either participate in a live simulation or in a virtual reality simulation using avatars	Medicine Nursing (undergraduate)
Brashers et al 2016 [61]	Measuring the Impact of Clinically Relevant Interprofessional Education on Undergraduate Medical and Nursing Student Competencies: A Longitudinal Mixed Methods Approach	Students participated in varying numbers of interprofessional workshops based on rapid response, end-of-life, chronic pediatric illness, and transitions of care for the cognitively impaired during a longitudinal study	Medicine Nursing (undergraduate)
King et al 2021 [62]	Use of an Academic Electronic Health Record with an Interprofessional Simulation for Advanced Practice Nursing Students	Interprofessional team rounds on two acutely ill standardized patients in a simulated ward, with teams either led by an advanced practice graduate nursing student or a medical student. An academic electronic health record was used to increase the complexity and realism of the simulation	Medicine Nursing (APRN) Occupational Therapy Pharmacy Physical Therapy Respiratory Therapy Speech Therapy Nutrition Services Social Work
Haber et al 2017 [63]	The Impact of Oral-Systemic Health on Advancing Interprofessional Education Outcomes	Students participated in an interprofessional clinical simulation and case study experience on the topic of oral-systemic health	Dentistry (DDS) Medicine Midwifery Nursing (graduate)
Karpa et al 2019 [64]	Geriatric Assessment in a Primary Care Environment: A Standardized Patient Case Activity for Interprofessional Students	An interprofessional simulation using standardized patients with a focus on geriatric assessment was developed and implemented for health professional students. Interprofessional communication was one of three dedicated educational objectives	Dental hygiene Dietetics and Nutrition Medicine Nursing (undergraduate) Occupational Therapy Pharmacy Physical Therapy
Lau et al 2019 [65]	Interprofessional Simulation-Based Advanced Cardiac Life Support Training: Video-Based Observational Study	Students completed a simulation-based training in Advanced Cardiac Life Support. Video recordings of the student teams were rated using the Clinical Teamwork and Communication and Teamwork Skills Assessment scales	Medicine Nursing (undergraduate)
Anderson et al 2019 [32]	Taking a Closer Look at Undergraduate Acute Care Interprofessional Simulations: Lessons Learnt	Medical, nursing, pharmacy, and operating department practitioners at the end of their respective educational programs were placed in teams to complete four high-fidelity interprofessional scenarios. Teams completed a pre-brief before the simulations, and a structured debrief after	Medicine Nursing (unspecified) Operating Department Practitioner Pharmacy

^a^Studies in order of the lowest Kirkpatrick’s Expanded Outcomes Typology identified

^b^Medicine includes Doctor of Medicine (MD) or Doctor of Osteopathic Medicine (DO), undergraduate

**Fig. 1:**
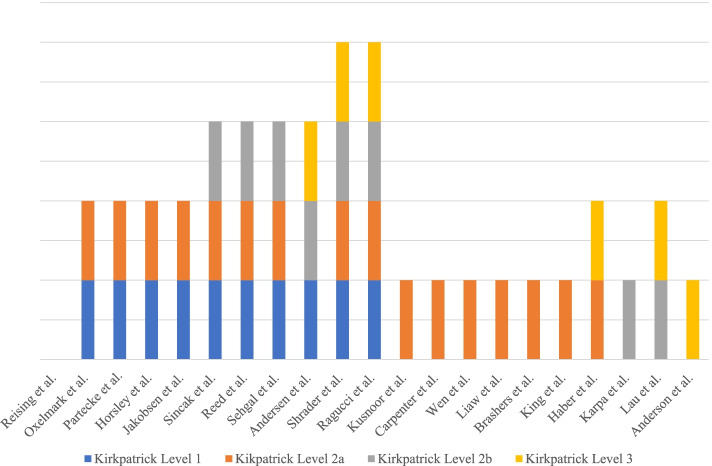
Kirkpatrick’s Level of Evaluation for included studies: Level 1: Learner’s reaction (Learners’ views on the learning experience and its interprofessional nature); Level 2a: Modification of attitudes/perceptions (Changes in reciprocal attitudes or perceptions between participant groups; changes in attitudes or perceptions regarding the value and/or use of team approaches to caring for a specific client group); Level 2b: Acquisition of knowledge/skills (Including knowledge and skills linked to interprofessional collaboration); Level 3: Behavioral change (Individuals’ transfer of interprofessional learning to their practice setting and their changed professional practice) (IOM 2015) [49–54, 57–61, 63–65]

The majority (76%, 16/21) of activities reported modification of attitudes/perceptions (Kirkpatrick Level 2a) as the primary learning outcome; 48% (10/21) reported assessment of learners’ reactions (Kirkpatrick Level 1); 38% (8/21) reported assessment of knowledge and/or skill acquisition (Kirkpatrick Level 2b); and 29% (6/21) reported assessment of behavioral change (Kirkpatrick Level 3) (Fig. 1).

In addition to IPEC competencies dedicated to interprofessional communication, authors also explicitly attempted to address IPEC competencies focused on teams/teamwork in 86% (18/21) of activities, followed in frequency by roles/responsibilities (48%, 10/21) and values/ethics for interprofessional practice (29%, 6/21). The majority (90%, 19/21) of activities captured quantitative data related to IPE learning outcomes using an assessment tool with established validity evidence in IPE (57%, 12/21), survey, or locally developed tool [66]. The majority (67%, 14/21) of included articles reported capturing qualitative data to inform IPE outcomes assessment, as well. Standardized communication tools such as Situation, Background, Assessment and Recommendation (SBAR), Identify, Situation, Background, Assessment and Recommendation (ISBAR) and TeamSTEPPS were utilized in 33% (7/21) of the simulation IPE activities.

Author-reported challenges included scheduling limitations when combining different health professional learners, time demands on faculty, geographic challenges due to different school locations, technologic complications with online activities, and lack of control groups to facilitate comparative analyses. Mismatches in learner cohort sizes resulted in disproportionate numbers of one type of learner compared to another in several simulation IPE activities, as well as mismatches of different types of learners according to learning level (e.g., novice, intermediate, advanced).

## Discussion

In this study, we found significant variability in terms of health-related topics taught and instruction methods employed during simulation IPE activities focused on interprofessional communication in simulation centers. However, high-fidelity simulations emerged as a predominant method of instruction, with the majority utilizing SPs. Standardized patients are considered the highest fidelity simulators and are used frequently to teach communication and interpersonal skills within undergraduate health professional curricula to improve transfer to the clinical learning environment [67, 68]. Standardized patients provide a clear benefit over role play in an authentic, yet ‘safe’ first experience in clinical interactions, while also providing professional feedback from the perspective of the patient [26, 69–71]. Performance feedback from SPs is considered a reliable source for assessing communication skills in educational programs [69, 71, 72]. Standardized patients are frequently used to increase the fidelity of interprofessional communication-focused simulation IPE activities, consistent with our findings. The addition of SPs can further increase the fidelity of simulation activities that do not currently utilize them; however, costs associated with utilizing SPs can be a limitation [26, 70].

We limited our focus to simulation IPE activities that were located in simulation centers based on the assumption that characteristics common to this setting – controlled environment, skilled staff, SPs, mannikins, and robust infrastructure/equipment – would inform efforts to target higher-level learning outcomes along the Kirkpatrick continuum. Interestingly, our results demonstrated that most simulation IPE targeted lower-level learning outcomes despite taking place in simulation centers. Furthermore, we postulate that 62% (13/21) of the simulation IPE activities identified in our study can be completed outside of a simulation center; thus, many of these simulation IPE activities can likely be transferred to classroom, hospital, or clinic settings.

Another interesting finding from our study is the identified lack of simulation IPE developed to span the entire continuum of Kirkpatrick levels (Fig. 1), with only 9.5% (2/21) of articles demonstrating this approach [55, 56]. The authors of these studies note challenges associated with this approach, including cost, logistics, schedules, faculty availability, and location of learners. A recommendation for achieving success in simulation IPE based on these two studies includes ensuring diversification of the types of modalities and/or pedagogical strategies employed, such as using video, telephone, workshops, and online simulations. By diversifying modalities and strategies, higher-level learning outcomes can be achieved with lower fidelity simulation IPE activities and less resources. In addition, less physical space is required for the activity at one given time and may not need to be located in a simulation center. Including video, telephone, or online simulations also eliminates the need for students to be geographically localized together [55].

Bok et al. described the characteristics of prevailing interprofessional communication programs for medical students in a scoping review from 2000–2018, identifying the indications and content of the programs, what training and evaluation methods each program used with the outcomes, and challenges experienced in 73 articles [38]. A content and thematic analysis was performed and themes/categories related to each of the four levels of Miller’s Clinical Assessment Framework/Pyramid were described [38, 73]. We chose to characterize activities in our review using Kirkpatrick’s model as it is used frequently in medical education and is recommended within consensus guidelines for quality IPE, though Kirkpatrick’s Expanded Outcomes Typology has been compared to Miller’s Pyramid [14, 74–76]. Similar to Bok et al., we found significant variability in the assessment of simulation IPE activities in our analysis, with the majority targeting learners’ reactions and modification of attitudes/perceptions, both considered lower-level outcomes. In another review, Abu-Rish et al. reported on qualitative, quantitative, and mixed method IPE studies published over five years that focused on IPE skills and/or competencies with an assessment of IPE effectiveness, which included 22 simulation IPE studies [36]. Abu-Rish et al. highlighted several similar patterns identified in our analysis; for example, assessments predominantly targeted lower-level learning outcomes and the majority of instructional designs featured one-time events [36].

Numerous IPE measurement tools, teamwork assessment tools, and simulation assessment tools used in activities identified in our review were utilized (Table 2). These assessment tools have documented validity evidence in IPE assessment, though we recognize that the validity of the tool is dependent on the context [66]. Studies that utilized one of these tools aimed to measure the same outcome of the original study of validation, such as attitudes or IPEC competencies, but did not always use the same groups of learners or setting. Some tools assessed simulation by observation to include IPE competencies but were not specific to IPE competencies. Our analysis validates much of what Bok et al., Abu-Rish et al., and others have reported, but Bok et al. and Abu-Rish et al. found that few programs utilized tools with established validity evidence in the assessment, whereas we found that the majority of simulation IPE activities used tools with established validity evidence [27, 36, 38]. Other studies have found that there is a lack of simulation IPE that is assessed with tools with validity evidence [27]. Our findings differ while simultaneously highlighting that tools with prior validity evidence should be used in a similar context and should continue to undergo the process of validation, thus adding to the literature [66].

**Table 2:** IPE measurement tools, teamwork assessment tools, and simulation assessment tools utilized by medical education simulation IPE articles

Attitudes Toward Health Care Teams Scale (ATHCT)
Clinical Teamwork Scale
Collaborative Behaviors Observational Assessment Tool (CBOAT)
Communication and Teamwork Skills
Interprofessional Collaborative Competencies Attainment Survey (ICCAS)
Interprofessional Socialization and Valuing Scale (ISVS)
Interprofessional Team Observation Feedback Tool
Modified Simulation Effectiveness Tool
Satisfaction with Simulation Experience Scale
Self-Efficacy Measure for Interprofessional Practice Competencies for Students
TeamSTEPPS Team Performance Observation Tool
Team Skills Scale (TSS)

The dearth of longitudinal simulation IPE activities in our study is consistent with what others have observed [36]. Longitudinal IPE activities with longitudinal follow-up of outcomes have shown improved interprofessional skills, communication, performance, and behaviors [39, 40, 47, 48]. More longitudinal IPE activities are needed in addition to longitudinal assessment to identify whether outcomes are long-term and if they ultimately lead to improved patient outcomes [36, 38, 47, 48]. Also congruent with our results were the difficulties and barriers to simulation IPE activities identified by authors, such as scheduling, mismatches of students at various levels of training, and lack of funding, faculty, staff and administrative support [36]. We agree there is significant diversity in the structure/content, objectives, and assessment of simulation IPE activities centered around interprofessional communication for medical students and a longitudinal approach to developing these activities should be prioritized [36, 38, 39].

The combination of modalities such as online activities and use of mannikins, SPs, and virtual reality in simulation IPE activities were limited in our review, as well as in the review by Bok et al. [38]. Benefits to hybrid activities include the elimination of logistical and resource barriers in a hybrid model to improve access to successful IPE [55, 62, 77]. Combining modalities in a longitudinal simulation IPE approach would also allow for repeated IPE interactions between learners with less dependence on space and time constraints, which would provide more opportunities for longitudinal IPE activities [39]. Students exposed to multiple IPE experiences across different settings have reported an increased impact on knowledge, skills, and attitudes than a single isolated IPE activity [55, 56, 78, 79]. By augmenting an SP encounter with a simulated electronic medical record review, for example, health professional learners can gain additional practical experience with added realism [62]. Our review documented the combined use of mannikins and SPs most frequently in hybrid activities; however, and importantly, we found that most of these hybrid IPE simulation could be completed outside of simulation centers and that they could be modified to attain higher-level learning outcomes along the Kirkpatrick continuum. IPE simulation activities should attempt to combine multiple teaching modalities for high-fidelity activities focused on interprofessional communication.

In our analysis, health professional learners identify the need for effective interprofessional communication. Simulation IPE, regardless of the type of modality used, can be used effectively to improve interprofessional communication based on the intended learning outcome and this seems to be independent of the location of the simulation. Reising et al. demonstrated that interprofessional communication improves performance [47]. This knowledge, combined with our results, should motivate medical educators to develop simulation IPE activities aimed at improving medical students’ capacity for team-based care through improved interprofessional communication [3, 8, 9, 47]. Based on our findings and recommendations of the Health Professions Accreditors Collaborative and the National Academy of Medicine, we also recommend greater use of the Kirkpatrick Expanded Outcomes Typology to drive the design and assessment of longitudinal simulation IPE activities that target higher-level learning outcomes, such as skill acquisition and behavior change [14, 41].

## Conclusion

This secondary analysis of a scoping review identified 21 articles published after 2016 that described simulation IPE activities involving medical learners in a simulation center and focused on interprofessional communication. While differences across these articles emerged regarding healthcare topics addressed, assessment tools utilized, learning outcomes measured, IPEC competencies targeted, and the types and combinations of health professional learners involved, several commonalities were identified that are instructive for medical educators throughout the world. We can draw from these commonalities and from those few studies included that have responded to calls from the IPE community to develop longitudinal IPE activities. To strengthen IPE simulation focused on interprofessional communication, we recommend that medical educators deliberately incorporate (1) hybrid instructional methods to bypass logistic hurdles, (2) longitudinal approaches to achieve higher-level learning outcomes, and (3) assessment tools with established validity evidence to measure those outcomes. Informed by our findings, this strategy will improve the skills and teamwork behaviors of medical students to improve patient care and outcomes.

## Supplementary Information

**Additional file 1. **PRISMA 2009 Flow Diagram.

## Data Availability

Not applicable.
