# Supplementary material for: Interprofessional communication in medical simulation: findings from a scoping review and implications for academic medicine
Source: BMC Med Educ. 2022 Mar 26;22:204. doi: 10.1186/s12909-022-03226-9 (PMC8962252; doi:10.1186/s12909-022-03226-9)
Supplement: Supplementary file 1 — Additional file 1. PRISMA 2009 Flow Diagram. [file 12909_2022_3226_MOESM1_ESM.doc]

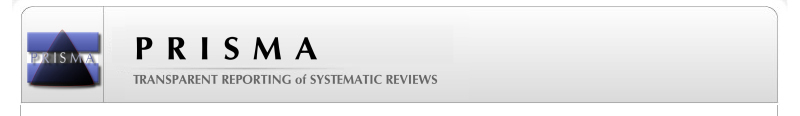
**PRISMA 2009 Flow Diagram**

**Screening**

**Included**

**Eligibility**

**Identification**

Records identified through database (PubMed, CINAHL, and ERIC) search (n = 2061)
(n = 2061)

Additional records identified through other sources
(n = 26)

Total duplicates removed
(n = 415)

Records screened
(n = 1672)

Records excluded
(n = 1371)

Full-text articles assessed for eligibility
(n = 301)

Full-text articles excluded
(n = 136)

(Incorrect population, incorrect study design, no simulation, no intervention, wrong publication type, wrong outcome, single discipline or student type, not a study, refined publication date 2016-present.

Studies included in qualitative synthesis
(n = 165)

Interprofessional communication secondary analysis applied

Studies included in secondary analysis
(n = 21)
